# Topical Tranexamic Acid Application to Facilitate Biopsy Acquisition in Endoscopic Nasopharyngeal Biopsy: A Prospective Case Series Analysis

**DOI:** 10.3390/jcm15062275

**Published:** 2026-03-17

**Authors:** Haldun Septar, Andra Iulia Suceveanu, Alina Doina Nicoara, Mihai Victor Lupascu, Alexandru Aristide Alexe, Iulia Cindea, Viorel Gherghina, Catalin Nicolae Grasa, Anca Pantea Stoian, Adrian Paul Suceveanu

**Affiliations:** 1 Department of Otorhynolaryngology, Faculty of Medicine, Ovidius University, 900527 Constanta, Romania; septa_seven@yahoo.com (H.S.); dr_lupa@yahoo.com (M.V.L.); alex.alexe93@gmail.com (A.A.A.); 2 Department of Gastroenterology, Faculty of Medicine, Ovidius University, 900527 Constanta, Romania; asuceveanu@yahoo.com; 3 Department of Internal Medicine, Faculty of Medicine, Ovidius University, 900527 Constanta, Romania; 4 Department of Intensive Care, Faculty of Medicine, Ovidius University, 900527 Constanta, Romania; iuliacindea@yahoo.com (I.C.); gherghina_v@yahoo.co.uk (V.G.); 5 Department of General Surgery, Faculty of Medicine, Ovidius University, 900527 Constanta, Romania; catalin.grasa@yahoo.com; 6 N.C. Paulescu National Institute of Diabetes, Nutrition and Metabolic Disease, Carol Davila University of Medicine and Pharmacy, 050474 Bucharest, Romania; ancastoian@yahoo.com

**Keywords:** tranexamic acid, nasopharyngeal carcinoma, biopsy, nasopharyngeal hemorrhage, topical antifibrinolytic therapy, hemostasis

## Abstract

**Background:** Nasopharyngeal carcinoma diagnosis requires endoscopic biopsy, but intraoperative hemorrhage frequently impairs visualization and compromises tissue sampling quality. This prospective case series evaluated topical tranexamic acid (TXA) as a hemostatic adjunct to improve biopsy conditions in suspected nasopharyngeal malignancy. **Methods:** Adults (≥18 years) with clinically/radiologically suspected nasopharyngeal tumors underwent pre-biopsy laboratory screening and exclusion of thromboembolic risk factors. After topical lidocaine anesthesia, a TXA-soaked cotton pledget was applied to the lesion for 10 min prior to forceps biopsy using 0° 4 mm endoscopy. Bleeding severity was graded pragmatically (minimal: ≤3 gauze pledgets; moderate: >3 or cauterization). Comparative analyses excluded rare diagnoses (*n* = 1). **Results:** Of 40 enrolled patients, 34 underwent biopsy (mean age 58.4 ± 12.3 years). All 34 biopsies (100%) yielded conclusive histopathological diagnoses. Adequate hemostasis was achieved in 97.1% (33/34), with minimal bleeding in 76.5% and moderate/massive in 23.5%. Non-keratinizing squamous cell carcinoma (44.1%) showed higher moderate bleeding rates than other diagnoses (Fisher’s exact *p* = 0.00035). Mean hospitalization was 1.79 ± 1.92 days, uniform across categories. No TXA-related adverse events occurred. **Conclusions:** Topical TXA provided safe, effective hemostasis during nasopharyngeal biopsy across diverse pathologies, achieving 100% diagnostic adequacy and short hospital stays. Controlled trials comparing TXA versus standard hemostatic techniques are warranted.

## 1. Introduction

Nasopharyngeal carcinoma (NPC) represents a significant global health burden with pronounced geographic variation. While endemic regions in Southeast Asia—particularly Southern China—report incidence rates of 25–50 per 100,000 males, Western populations demonstrate rates below 1 per 100,000. Romania presents an increasingly concerning epidemiological trend, with a 196% rise in age-standardized incidence between 1990 and 2019 [1,2].

The etiopathogenesis of NPC reflects a complex interplay of genetic, environmental, and infectious factors. Epstein–Barr virus remains the central pathogenic driver, particularly in endemic regions dominated by EBV-associated undifferentiated carcinoma. Well-established risk factors include tobacco use (2–6 fold increased incidence), alcohol consumption, nitrosamine-rich diets, occupational exposures, and familial clustering, supporting a hereditary component. NPC occurs across all age groups with a characteristic peak between 40 and 60 years of age [3].

Early diagnosis of NPC remains challenging despite advances in endoscopic visualization. Clinical presentation is frequently nonspecific, with initial symptoms often misattributed to benign conditions, resulting in substantial diagnostic delays averaging 4–6 months in some series. The disease’s rarity in non-endemic populations contributes to diagnostic uncertainty. Critically, the lack of sensitive and specific clinical biomarkers necessitates tissue confirmation via endoscopic biopsy for definitive diagnosis [1].

The nasopharynx presents significant technical challenges for endoscopic tissue sampling due to limited accessibility, high vascularity, and confined anatomical space. Hemorrhage during endoscopic biopsy impairs visualization and compromises specimen quality, resulting in suboptimal diagnostic yields that directly impact clinical outcomes and delay appropriate therapy. Even modest bleeding can severely obscure the surgical field, increasing the risk of tissue sampling error or incomplete specimen collection [4].

Traditional hemostatic approaches—including instillation of iced saline with vasoconstrictors (xylometazoline, epinephrine), mechanical compression, thermal cautery, and hemoclips—remain suboptimal for tissue sampling procedures. Biopsy itself interrupts hemostasis and reinitiates bleeding, while thermal hemostasis may induce tissue artifact that compromises histopathological examination. In clinical practice, failed hemostasis may require nasal packing or electrocauterization, both associated with increased morbidity [4,5].

Tranexamic acid (TXA) is a synthetic lysine analogue that competitively inhibits fibrinolysis by blocking plasminogen activation and preventing fibrin degradation [6]. The drug demonstrates a short half-life of 2–3 h with 95% renal elimination. Topical application is particularly advantageous for endoscopic procedures, delivering high local concentrations while achieving minimal systemic absorption and thereby reducing theoretical risks of thromboembolic complications [7].

Evidence across multiple surgical specialties demonstrates TXA’s hemostatic efficacy. In otolaryngological procedures, meta-analytic evidence shows that tranexamic acid reduces intraoperative blood loss during endoscopic sinus surgery, improves surgical field quality, and shortens operative time [8]. For epistaxis management, topical tranexamic acid reduces rebleeding and achieves hemostasis faster than alternative topical agents [5,9]. A prospective case series of bronchoscopic intratumoral tranexamic acid injection (250–500 mg) demonstrated successful hemorrhage control during forceps biopsy of hypervascular endobronchial tumors, enabling acquisition of multiple quality specimens with histopathological diagnoses established in all cases and no adverse effects reported [10].

Although there is a substantial body of literature on nasopharyngeal hemorrhage in the setting of radiotherapy [11,12,13] or advanced disease, systematic quantification of bleeding specifically associated with nasopharyngeal biopsy remains scarce. Most series of nasopharyngeal masses describe endoscopic biopsy as generally safe and refer only briefly to “minor bleeding”, without reporting blood-loss volumes or using standardized severity scales. In addition, surgical atlases and ENT procedural manuals emphasize another important limitation of biopsy: sampling from an incorrect region of the nasopharynx may yield only reactive lymphoid hyperplasia, thereby delaying the diagnosis of carcinoma and necessitating repeat procedures [14,15].

Despite extensive evidence supporting TXA’s hemostatic efficacy in related endoscopic procedures, the specific application of topical tranexamic acid to improve diagnostic yield during endoscopic nasopharyngeal biopsy of nasopharyngeal masses remains understudied. Topical TXA application represents a rational, cost-effective, and safe intervention with potential to enhance hemostatic control, reduce procedure-related bleeding complications, improve specimen quality, and ultimately improve diagnostic yield.

This prospective case series was designed to assess the efficacy and safety of topical tranexamic acid applied to nasopharyngeal tumoral masses prior to endoscopic forceps biopsy in patients suspected of nasopharyngeal malignancy. This procedure is not part of standard care at our institution and was performed for the purpose of this research.

The study protocol received prior approval from the Institutional Ethics Committee, and all procedures were conducted in accordance with the Declaration of Helsinki and current European Union regulations on clinical research ethics.

## 2. Methods

The study was designed as a prospective case series study and carried out at a single center. Patients aged 18 years or older who were suspected of having a nasopharyngeal tumor based on clinical signs or symptoms, computed tomography or MRI findings were enrolled to undergo endoscopical forceps biopsy.

Before the procedure, all patients underwent screening that included assessments of liver and kidney function, complete blood counts, and coagulation studies (platelet count, prothrombin time, and activated partial thromboplastin time).

Patients were excluded if they were medically unfit to undergo endoscopy, unwilling to provide written informed consent, had a history or risk factors for thrombosis, active thromboembolic disease, subarachnoid hemorrhage, or known/suspected bleeding disorders. Additional exclusion criteria included a platelet count < 150,000/mm^3^ and an international normalized ratio (INR) > 1.3.

After three puffs of 10% lidocaine spray to each nostril for local anesthesia of the nasal fossa, transnasal endoscopy of the rhinopharynx using a 0° 4 mm diameter rigid endoscope was performed to identify and visualize the tumoral mass. Two Transamine ampoules (50 mg/mL TXA, TEVA Ltd., Istanbul Turkey) were drawn into a metal bowl and used to soak a cotton pledget, which was then placed in direct contact with the tumor surface in the nasopharynx for 10 min. Multiple biopsy specimens were then obtained from the lesion using standard cupped biopsy forceps. All patients were followed up for 5–7 days after the procedure.

Intraoperative bleeding was categorized pragmatically as minimal or moderate based on the number of unfolded 5 × 5 cm, 8-ply ENT gauze pledgets (absorption capacity: 3.0–3.8 mL per pledget [16,17,18]) required for haemostasis at the nasopharyngeal level.

Minimal bleeding was defined as haemorrhage successfully controlled with ≤3 pledgets (≤9.0–11.4 mL total blood loss) without need for additional local interventions beyond suction. Moderate bleeding was defined as haemorrhage requiring >3 pledgets (≤9.0–11.4 mL total blood loss) and/or additional local interventions such as endoscopic monopolar cauterization. Massive bleeding was defined as haemorrhage necessitating both endoscopic cauterization and anterior–posterior nasal packing to achieve stable haemostasis.

Data were analyzed using IBM SPSS Statistics version 29.0 (IBM Corp., Armonk, NY, USA). Continuous variables were reported as mean ± standard deviation (SD) or median (interquartile range [IQR]) and categorical variables as frequencies and percentages. Comparative statistical analyses were restricted to the six diagnostic categories with ≥2 cases each (total *n* = 31 patients) to ensure adequate statistical power and within-group variance:—Age and length of hospital stay (LOS): One-way analysis of variance (ANOVA) followed by Tukey’s honestly significant difference (HSD) post hoc test for multiple pairwise comparisons—Association between diagnosis and bleeding severity: Pearson’s chi-square test (minimum expected cell count ≥ 5). Rare diagnostic categories (sarcoma, plasmacytoma, adenocarcinoma; *n* = 1 each) were excluded from comparative analyses due to the absence of within-group variance but included in descriptive cohort characteristics. A two-sided *p*-value < 0.05 was considered statistically significant. Graphs display error bars representing mean ± SD, with statistical significance indicated by asterisks in figure legends (*p* < 0.01, *p* < 0.05).

## 3. Results

Between October 2021 and November 2025, 40 patients were prospectively enrolled in this study. Strict application of predefined inclusion and exclusion criteria resulted in 34 eligible patients (mean age ± SD, 58.4 ± 12.3 years; range, 36–90 years) who underwent the biopsy procedure. All 34 biopsies yielded conclusive histopathological diagnoses (100% diagnostic adequacy rate), with no nondiagnostic or inadequate specimens reported. This high rate of diagnostically useful biopsies was obtained under TXA-assisted haemostatic conditions, although direct comparison with alternative haemostatic techniques was not performed.

Table 1 and Table 2 summarizes age, sex distribution, and key pre-procedural laboratory parameters for all 34 patients (descriptive) and 31 patients in 6 major diagnostic groups (comparative analysis) who underwent endoscopic nasopharyngeal biopsy with topical tranexamic acid application. Continuous variables are presented as mean ± standard deviation and categorical variables as *n* (%). No statistically significant differences were observed in baseline characteristics between diagnostic categories unless otherwise indicated.

Non-keratinizing squamous cell carcinoma represented the predominant diagnosis (44.1%), occurring mainly in older adults (mean age 57.9 years) and showing comparable rates of minimal and moderate intraoperative bleeding. Lymphomas accounted for 20.6% of cases, with a broader age range and exclusively minimal bleeding. Inflammatory lesions (8.8%) and adenoiditis (5.9%) were less frequent and generally associated with short hospital stays and minimal hemorrhage. Keratinizing squamous cell carcinoma (SCC) accounted for 5.9% of cases in the studied cohort. These tumors were identified exclusively in older patients, with a mean age of 65.5, supporting the well-established epidemiological association between keratinizing histological subtype and advanced age.

Rare histopathological entities—including sarcoma, plasmacytoma, and adenocarcinoma (*n* = 1 each; 8.8% of the total cohort)—were excluded from comparative statistical analyses because of their very low frequency and the inherent lack of analytical power. Overall, the cohort had a mean age of 58.4 years and a mean hospital stay of 1.79 days, with a balanced sex distribution. Minimal bleeding occurred in 76.5% of procedures, while moderate bleeding was noted in 20.6%, confirming the generally safe intraoperative profile across diagnostic categories.

### 3.1. Diagnostic Distribution and Age-Related Patterns

Squamous cell carcinoma (SCC) non-keratinized represented the predominant diagnosis (44.1%, *n* = 15), consistent with epidemiological patterns in regions with high nasopharyngeal malignancy burden. Notably, lymphomas accounted for 20.6% of cases (*n* = 7), which is a higher proportion than typically reported in Western countries but comparable to cohorts from geographic regions with increased prevalence of Epstein–Barr virus-associated lymphomas [14]. The remaining diagnoses (inflammatory lesions, adenoiditis, other carcinomas, keratinized SCC, sarcoma, plasmacytoma, and adenocarcinoma) collectively represented 35.3% of the cohort, reflecting the heterogeneous nature of nasopharyngeal pathology encountered in tertiary otolaryngology centers. Three rare entities—sarcoma, plasmacytoma, and adenocarcinoma—each occurred in a single patient (*n* = 1 per category) and were therefore excluded from comparative statistical analyses, although they are retained in the descriptive summary of the cohort.

**Figure 1:**
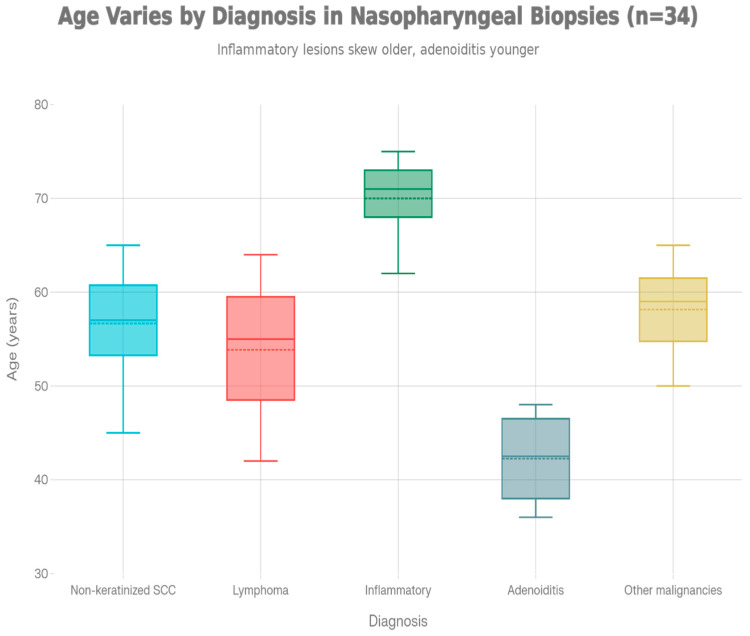
Age at presentation by histopathological diagnosis (*n* = 34). Box plots depict the age distribution for each diagnostic category (x-axis: diagnosis; y-axis: age in years). Inferential analysis was restricted to the six diagnostic groups with at least two patients each (*n* = 31); one-way ANOVA did not show a statistically significant difference in age between diagnoses (F(5,25) = 1.77, *p* = 0.155).

When the analysis was restricted to the six diagnostic categories with at least two patients per group (total *n* = 31), one-way ANOVA did not demonstrate a statistically significant association between diagnosis and age at presentation (F(5,25) = 1.77, *p* = 0.155). Descriptively, inflammatory lesions (70.0 ± 13.0 years) and keratinizing SCC (65.5 ± 2.1 years) tended to occur in older patients, whereas adenoiditis (47.0 ± 11.3 years) and other benign pathology were observed in comparatively younger individuals. Given the limited sample size in several categories, these apparent age differences should be interpreted as exploratory trends rather than definitive statistical findings (Figure 1).

This age-diagnosis correlation likely reflects the natural history of nasopharyngeal pathology, where inflammatory and degenerative processes predominate in older populations, while adenoid hypertrophy and benign tumors manifest in younger cohorts. These findings align with established literature demonstrating age-dependent differences in oncologic and inflammatory presentations within the head and neck region.

### 3.2. Tranexamic Acid Efficacy Across Diagnostic Categories

A principal finding of this investigation was the diagnosis-independent efficacy of tranexamic acid in controlling nasopharyngeal hemorrhage. Adequate intraoperative hemostasis was achieved in 33 of 34 patients (97.1%), with only one individual requiring additional antero-posterior packing. Because of this very low failure rate, formal hypothesis testing of TXA success across individual diagnostic categories was not considered statistically appropriate; TXA appeared effective in all major diagnostic groups. This observation is particularly noteworthy given the marked heterogeneity of vascular physiology across different diagnostic entities.

**Figure 2:**
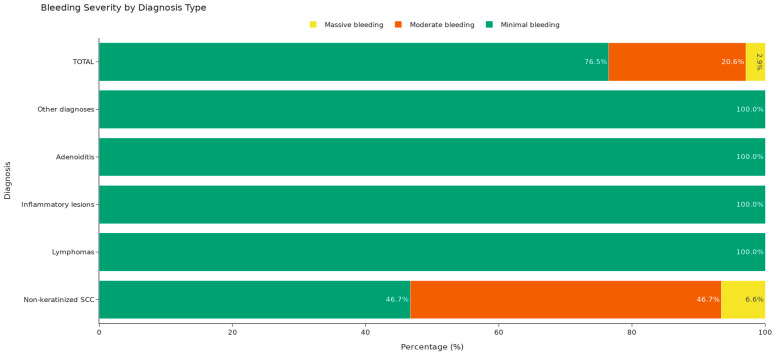
Intraoperative bleeding severity according to diagnosis. This stacked bar graph illustrates the proportion of patients with minimal and moderate intraoperative bleeding in each diagnostic category (x-axis: diagnostic groups; y-axis: proportion of procedures, %). Minimal and moderate bleeding are color-coded and defined in the main text.

Bleeding severity was further analysed by classifying procedures as minimal, moderate, or massive. Overall, minimal bleeding occurred in 76.5% of procedures (26/34), moderate bleeding in 20.6% (7/34), and massive bleeding in 2.9% (1/34), the latter observed in a single patient with non-keratinizing squamous cell carcinoma (SCC). All patients with lymphoma (7/7), inflammatory lesions (3/3), adenoiditis (2/2), and other benign or non-SCC malignant pathology (5/5) had only minimal bleeding under TXA treatment (Figure 2).

To address the small sample size in several diagnostic categories, inferential analysis was simplified to a 2 × 2 comparison of non-keratinizing SCC versus all other diagnoses, with bleeding severity divided as minimal versus non-minimal (moderate or massive). In this analysis, non-minimal bleeding occurred in 8 of 15 non-keratinizing SCC cases (53.3%) but in 0 of 19 procedures performed for other diagnoses, and Fisher’s exact test confirmed a statistically significant difference (*p* = 0.00035). Thus, while TXA provided effective overall hemostasis in virtually all patients, non-keratinizing SCC was more likely to be associated with moderate or massive bleeding compared with other pathological entities.

These findings are consistent with the known biology of non-keratinizing SCC, which often demonstrates aggressive infiltrative growth and pronounced neo-angiogenesis, and suggest that such tumors may generate more robust bleeding despite antifibrinolytic therapy. Nevertheless, the achievement of adequate hemorrhage control in all but one non-keratinizing SCC case supports the use of TXA as a highly effective first-line adjunct even in this high-risk subgroup.

### 3.3. Hospitalization Duration and Clinical Management

**Figure 3:**
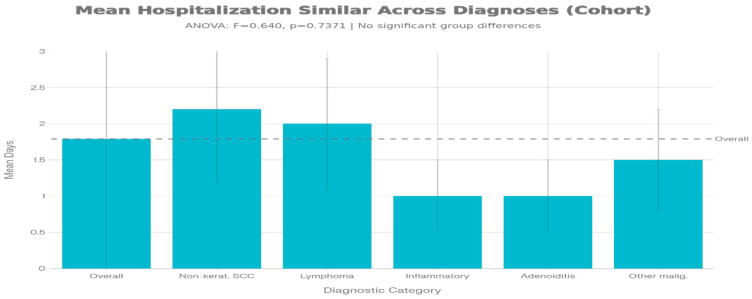
Hospitalization duration by diagnostic category. This figure presents mean length of hospital stay for each diagnostic group (x-axis: diagnostic categories; y-axis: hospital stay in days). Bars represent mean values with error bars indicating standard deviation. Inferential analysis was restricted to the six diagnostic categories with at least two patients per group (*n* = 31); one-way ANOVA demonstrated no statistically significant differences in LOS between diagnoses (F(5,25) = 0.46, *p* = 0.80).

A secondary finding of clinical relevance was the absence of significant variation in length of hospitalization across diagnostic categories (F = 0.640, *p* = 0.7371). Mean hospitalization duration ranged from 0.5 to 2.2 days across major diagnostic groups, with an overall cohort mean of 1.79 ± 1.92 days. This narrow range is remarkable and suggests that institutional protocols for nasopharyngeal pathology management have achieved standardization that effectively addresses the acute hemorrhage risk regardless of underlying diagnosis. Non-keratinized SCC patients, despite being the most common and potentially most challenging group, required a mean hospitalization of 2.20 days, only marginally longer than lymphoma patients (2.00 days) and inflammatory cases (1.00 days) (Figure 3).

This finding has important implications for healthcare resource allocation and cost optimization, indicating that expected hospitalization costs should be similar across diagnosis types. The standardized and brief hospitalization pattern observed in our cohort reflects the efficacy of TXA in enabling rapid hemostasis control and early patient discharge. Traditional management of nasopharyngeal hemorrhage, particularly in malignant disease, frequently necessitated prolonged hospitalization, repeated interventions, or surgical hemostasis procedures such as arterial ligation. The current series demonstrates that, using local hemostatic measures (such as topical TXA-soaked gauze packing), most patients can be managed effectively with brief observation periods and safe discharge within 1–2 days. This represents a significant advancement in the conservative management of nasopharyngeal hemorrhage.

Our findings support several evidence-based recommendations for the management of nasopharyngeal pathology with hemorrhage across the diagnostic spectrum. First, tranexamic acid should be considered a standardized first-line pharmacologic intervention for all patients presenting with nasopharyngeal hemorrhage, regardless of underlying diagnosis. The diagnosis-independent efficacy (97.1% overall success) and excellent safety profile make TXA an appropriate choice even before definitive diagnostic confirmation. This approach is particularly valuable in the emergency setting, where rapid hemostasis is required prior to completion of diagnostic workup. Second, while all diagnostic categories respond well to TXA, non-keratinized SCC patients may warrant closer monitoring for adequate response, given the slightly lower excellent response rate (46.7% vs. 100% for other diagnoses). Clinicians should be prepared to escalate intervention in this population if moderate rather than minimal bleeding persists after initial TXA administration. Potential escalation strategies might include repeated TXA dosing, prolonged local application of hemostatic agents, or selective arterial embolization in cases of massive hemorrhage refractory to pharmacologic management. Third, the consistent brief hospitalization pattern (mean 1.79 days across diagnoses) supports implementation of expedited discharge protocols for appropriately selected patients with nasopharyngeal hemorrhage. However, such protocols should incorporate appropriate safeguards, including clear outpatient follow-up instructions, consideration of patient reliability and access to emergency care, and assessment of hemostasis stability prior to discharge.

## 4. Discussion

The present case series demonstrates that topical tranexamic acid achieves consistent, diagnosis-independent hemostatic efficacy (97.1% overall success) in patients undergoing endoscopic nasopharyngeal biopsy for evaluation of nasopharyngeal pathology. These findings extend the growing body of evidence supporting TXA’s hemostatic utility in otolaryngological procedures.

Our results support topical tranexamic acid as a standardized first-line pharmacologic intervention for nasopharyngeal hemorrhage management, regardless of underlying diagnosis. The excellent efficacy and benign safety profile demonstrated in our cohort, combined with the absence of serious adverse events, make TXA an appropriate choice for rapid hemostasis even before definitive diagnostic confirmation. This approach is particularly valuable in emergency settings where rapid bleeding control is required.

While all diagnostic categories responded well to TXA, non-keratinized SCC patients demonstrated a slightly lower minimal bleeding rate (46.7% vs. 100% for other diagnoses). This observation may reflect the enhanced neo-angiogenesis and abnormal vessel architecture characteristic of malignant tumors. Nevertheless, the achievement of adequate bleeding control in 93.3% of non-keratinized SCC cases indicates excellent clinical efficacy. Clinicians managing these patients should be aware of the potential for moderate rather than minimal bleeding and prepared to escalate intervention if necessary.

The consistent brief hospitalization pattern (mean 1.79 days across diagnoses) supports implementation of expedited discharge protocols for appropriately selected patients with nasopharyngeal hemorrhage. However, such protocols should incorporate appropriate safeguards, including clear outpatient follow-up instructions, assessment of patient reliability and access to emergency care, and verification of hemostasis stability prior to discharge.

Our 97.1% efficacy rate compares favorably to historical data on conventional hemostatic management of nasopharyngeal hemorrhage, for which success rates typically range from 70–90% [19]. The high overall control of bleeding across most diagnostic entities is compatible with an important contribution of fibrinolytic activation to nasopharyngeal hemorrhage that can be targeted by antifibrinolytic therapy, but the present clinical data do not allow us to demonstrate a single unifying pathophysiological mechanism. Moreover, although TXA achieved adequate haemostasis in almost all patients, bleeding severity was more often moderate or massive in non-keratinizing SCC than in other diagnoses, indicating that its efficacy is not completely diagnosis-independent and may still be modulated by underlying tumor biology and vascular characteristics [20].

### Limitations and Methodological Considerations

Several limitations warrant acknowledgment. The small sample size for some diagnostic categories (*n* = 1–2 for rare diagnoses such as sarcoma, plasmacytoma, and adenocarcinoma) limits statistical power for group-specific comparisons and generalizability to these uncommon presentations. The lack of a control group receiving alternative hemostatic interventions prevents direct comparative assessment of TXA superiority, though our results compare favorably to historical data.

The absence of randomization introduces potential selection bias. Additionally, the retrospective nature of some aspects of data collection may result in incomplete documentation regarding exact hemorrhage quantification or precise TXA dosing timing.

Third, intraoperative bleeding severity was graded using a pragmatic, operator-dependent definition based on gauze usage rather than a validated surgical field scale such as the Boezaart score. Gauze size, suction technique and individual surgeon thresholds may introduce subjectivity. Future studies should incorporate standardized, validated haemorrhage grading scales and, where feasible, quantitative measures of blood loss. (suction canister gravimetry, hemoglobin dilution).

Although our investigation did not include a concurrent control group managed with conventional haemostatic strategies, several lines of evidence from related otorhinolaryngologic settings support a potential advantage of tranexamic acid over traditional measures. Randomized controlled trials in anterior epistaxis have demonstrated that the topical application of injectable tranexamic acid achieves more rapid hemostasis, higher rates of bleeding control within 10–30 min, and lower early rebleeding rates compared with standard anterior nasal packing or vasoconstrictor-based regimens [5,21,22,23]. Furthermore, meta-analytic data from endoscopic sinus surgery indicate that topical or systemic tranexamic acid reduces intraoperative blood loss, improves endoscopic field visibility, and shortens operative time relative to control interventions [24,25,26]. While these studies are conducted in different anatomical and procedural contexts, they collectively provide biologic plausibility and indirect clinical support for the use of tranexamic acid as a superior haemostatic adjunct compared with traditional techniques such as vasoconstrictor instillation, cauterization, or packing; nonetheless, our case series cannot replace a dedicated head-to-head comparison in nasopharyngeal tumour biopsy, which remains a key objective for future controlled trials.

## 5. Conclusions

This analysis of 34 patients with nasopharyngeal hemorrhage across nine diagnostic categories shows that tranexamic acid provides consistent, high efficacy in hemorrhage control regardless of underlying pathology. Although age at presentation differs by diagnosis, hospitalization duration and treatment response do not. These results support tranexamic acid’s promising potential as an adjunct for nasopharyngeal hemorrhage, particularly in regions with a high burden of nasopharyngeal malignancy. The uniform clinical response across diverse entities suggests a common pathophysiological pathway through which antifibrinolytic therapy corrects the core hemostatic disturbance in nasopharyngeal hemorrhage.

## Figures and Tables

**Table 1:** Baseline demographic and clinical characteristics of the study cohort.

Diagnosis	*n*	%	Age Mean ± SD	Age Range	LOS Mean ± SD	LOS Range	Male/Female	Minimal Bleed%	Moderate Bleed%	Massive Bleed%
SCC Non-keratinized	15	44.10%	57.9 ± 7.2	45–71	2.20 ± 2.01	0–7	12/3	46.70%	46.70%	6.7%
Lymphoma	7	20.60%	56.7 ± 15.6	36–77	2.00 ± 2.38	0–6	3/4	100.00%	0.00%	0.00%
Inflammatory	3	8.80%	70.0 ± 13.0	62–85	1.00 ± 1.00	0–2	1/2	100.00%	0.00%	0.00%
Adenoiditis	2	5.90%	47.0 ± 11.3	39–55	2.00 ± 2.83	0–4	0/2	100.00%	0.00%	0.00%
Carcinoma (other)	2	5.90%	48.0 ± 12.7	39–57	0.50 ± 0.71	0–1	2/0	100.00%	0.00%	0.00%
SCC Keratinized	2	5.90%	65.5 ± 2.1	64–67	1.00 ± 0.00	1–1	0/2	100.00%	0.00%	0.00%

**Table 2:** Rare nasopharyngeal tumor diagnoses and clinical characteristics of the study cohort.

Diagnosis	*n*	%	Age	LOS	Sex	Bleeding
* Sarcoma	1	2.9%	48	0	F	Minimal
* Plasmocytoma	1	2.9%	51	0	F	Minimal
* Adenocarcinoma	1	2.9%	90	4	M	Minimal

* Rare diagnoses excluded from comparative statistical analyses (*n* = 1 precludes within-group variance calculation).

## Data Availability

The data generated in the present study may be requested from the corresponding authors.
